# Features of CFTR mRNA and implications for therapeutics development

**DOI:** 10.3389/fgene.2023.1166529

**Published:** 2023-04-24

**Authors:** JaNise J. Jackson, Yiyang Mao, Tyshawn R. White, Catherine Foye, Kathryn E. Oliver

**Affiliations:** ^1^ Department of Pediatrics, Emory University School of Medicine, Atlanta, GA, United States; ^2^ Center for Cystic Fibrosis and Airways Disease Research, Emory University and Children’s Healthcare of Atlanta, Atlanta, GA, United States

**Keywords:** CFTR, mRNA stability, synonymous single nucleotide polymorphism, suppressor tRNA, miRNA, peptide nucleic acid (PNA), antisense oligonucleotide, readthrough compound

## Abstract

Cystic fibrosis (CF) is an autosomal recessive disease impacting ∼100,000 people worldwide. This lethal disorder is caused by mutation of the *CF* *transmembrane conductance regulator (CFTR)* gene, which encodes an ATP-binding cassette-class C protein. More than 2,100 variants have been identified throughout the length of *CFTR*. These defects confer differing levels of severity in mRNA and/or protein synthesis, folding, gating, and turnover. Drug discovery efforts have resulted in recent development of modulator therapies that improve clinical outcomes for people living with CF. However, a significant portion of the CF population has demonstrated either no response and/or adverse reactions to small molecules. Additional therapeutic options are needed to restore underlying genetic defects for all patients, particularly individuals carrying rare or refractory *CFTR* variants. Concerted focus has been placed on rescuing variants that encode truncated CFTR protein, which also harbor abnormalities in mRNA synthesis and stability. The current mini-review provides an overview of CFTR mRNA features known to elicit functional consequences on final protein conformation and function, including considerations for RNA-directed therapies under investigation. Alternative exon usage in the 5′-untranslated region, polypyrimidine tracts, and other sequence elements that influence splicing are discussed. Additionally, we describe mechanisms of CFTR mRNA decay and post-transcriptional regulation mediated through interactions with the 3′-untranslated region (e.g. poly-uracil sequences, microRNAs). Contributions of synonymous single nucleotide polymorphisms to CFTR transcript utilization are also examined. Comprehensive understanding of CFTR RNA biology will be imperative for optimizing future therapeutic endeavors intended to address presently untreatable forms of CF.

## Introduction

**FIGURE 1:**
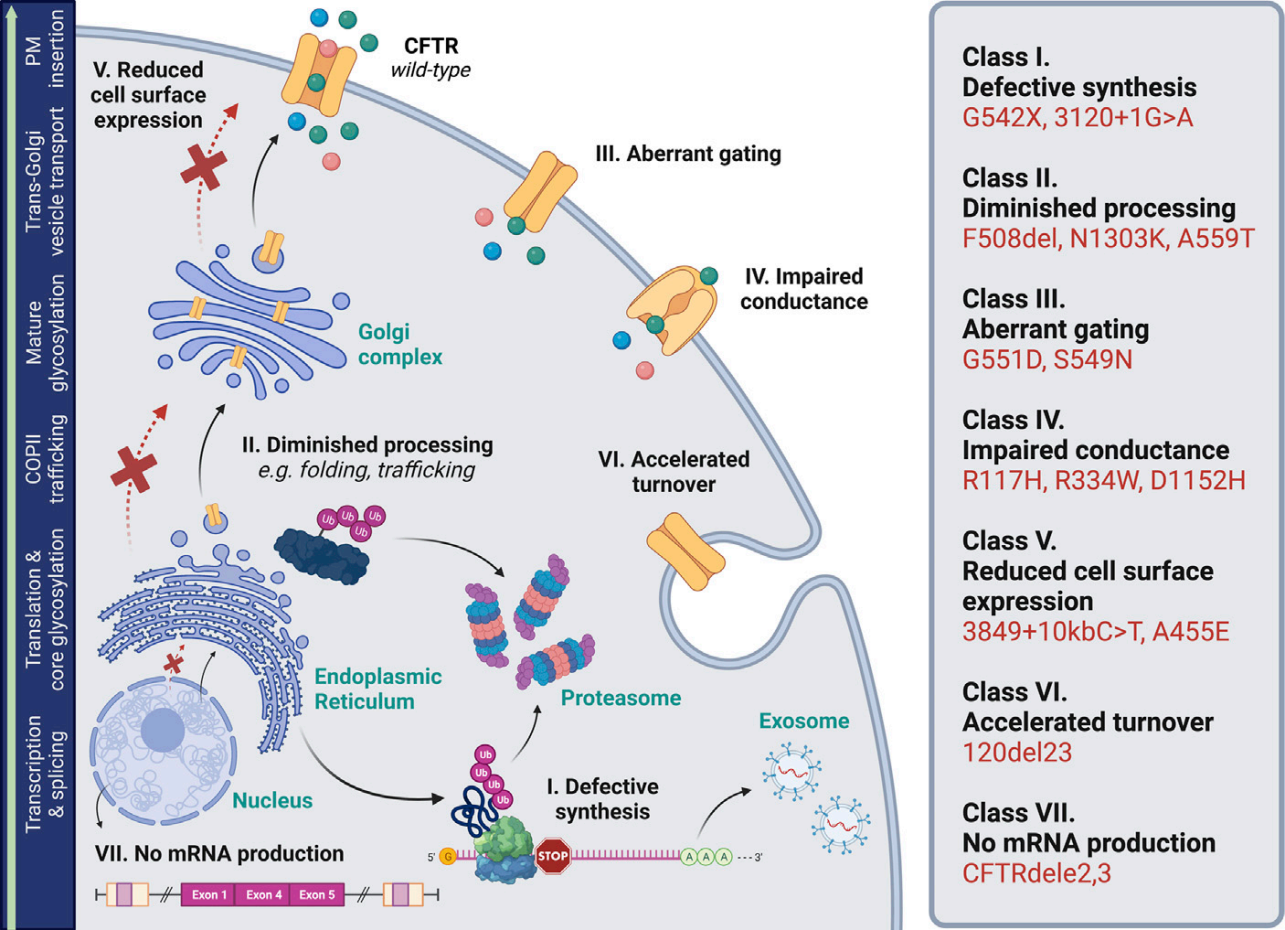
Biosynthetic pathway of CFTR. This ATP-binding cassette class-C transporter facilitates efflux of chloride (green molecule), bicarbonate (pink molecule), and water (blue molecule) across the plasma membrane (PM). Biogenesis encompasses transcription and splicing in the nucleus; translation, folding, and core glycosylation at the endoplasmic reticulum; transport to and through the Golgi, including further glycosylation and other post-translational modifications; and vesicular delivery to the cell surface. *CFTR* variants disrupt one or more steps in this pathway, and thus, are categorized into seven sub-classes based on molecular features (De Boeck and Amaral, 2016; Cottrill et al., 2020). These include defects in: (I) protein synthesis (e.g., nonsense codons, splice variants); (II) processing (e.g., folding abnormalities); (III) channel gating; (IV) ion conductance through a malformed pore; (V) expression at the cell surface (defects in trafficking are denoted with a red “X”); (VI) plasma membrane stability and turnover; and (VII) mRNA production. Abbreviations: coat protein complex II (COPII); ubiquitin (Ub); premature termination codon (“stop”). Image was generated using BioRender.

Cystic fibrosis (CF) is a monogenic disorder caused by loss-of-function of the CF transmembrane conductance regulator (CFTR), a chloride and bicarbonate channel expressed in respiratory, gastrointestinal, and exocrine tissues (among others) (Collins, 1992; Marson, 2018). With more than 2,100 disease-associated variants reported in *CFTR* (Sosnay et al., 2013; The Clinical and Functional TRanslation of CFTR CFTR2, 2021; Cystic Fibrosis Mutation Database, 2022), these variants are categorized by molecular attributes such as little-to-no transcript and/or polypeptide production, decreased protein maturation, or impaired channel gating (Oliver et al., 2017; Ensinck and Carlon, 2022) (Figure 1). Recent advancements in CF therapeutics have led to clinical approval of small molecules (“modulators”) primarily designed to rescue defects in CFTR folding or trafficking, otherwise termed “processing” variants. While at the forefront of personalized medicine, significant portions of the global CF population do not benefit from these modulators based on *CFTR* genotype (McGarry and McColley, 2021). Thus, there remains a critical need for efficacious treatment strategies to address presently off-label variants. Increasing evidence places CFTR mRNA quality and utilization as a key contributor to patient heterogeneity and pharmacologic responsiveness (Clarke et al., 2021; Rauscher et al., 2021; Bengtson et al., 2022). In this brief review, we summarize ways in which mRNA structure, stability, and abundance robustly influence CFTR functional expression. We also highlight new CF treatment paradigms targeted at the level of RNA.

### Features within the CFTR 5′-untranslated region (UTR)

**FIGURE 2:**
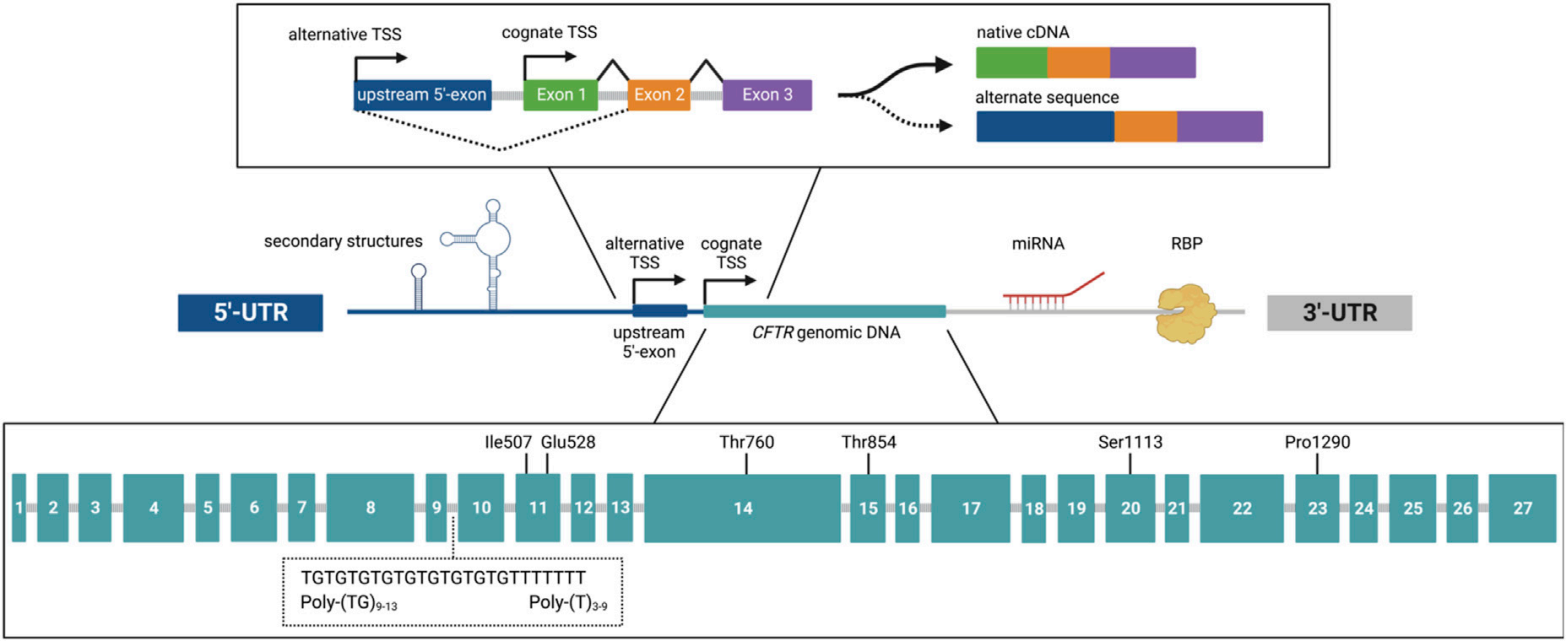
Major contributors to CFTR mRNA structure, abundance, and utilization. In the 5′-untranslated region (UTR), RNA secondary structures inhibit translation initiation and mRNA stability. Upstream 5′-exons introduced by an alternative transcriptional start site (TSS) demonstrate skipping of exon-1 *via* direct splicing to exon-2 (upper panel). The 3′-UTR facilitates enhanced or reduced CFTR mRNA expression through interaction of RNA-binding proteins (RBPs) with cytosine repeats, poly-uracil tracts, or AUUUA motifs. Engagement of miRNAs with the 3′-UTR also diminishes CFTR transcript abundance. Within the gene body, synonymous single nucleotide polymorphisms at residues Ile507, Glu528, Thr760, Thr854, Ser1113, and Pro1290 confer alterations to CFTR mRNA secondary structure, splice forms, and translation efficiency (lower panel). In addition, extended repeats of thymidine (9T) and/or thymidine-guanine (13TG) in intron 9 induce alternative splicing and skipping of exon-10. Image was generated using BioRender.

Intron excision and exon ligation are essential steps in pre-mRNA processing known as splicing (Ben-Dov et al., 2008). Through this mechanism, it has been well established that different patterns of intron removal can lead to diverse mRNA sequences and protein products originating from a single gene. A contributor to variable CFTR splice forms encompasses alternative exon usage within the 5′-UTR (Figure 2). In human CFTR, upstream 5′-exons generated from alternative transcriptional start sites demonstrate direct splicing to exon-2, effectively skipping the exon-1 (Chou et al., 1991; Yoshimura et al., 1991; Koh et al., 1993; Mouchel et al., 2003; Davies et al., 2004). Resulting transcripts exhibit Gibbs free energies reflective of stable RNA secondary structures. Such features would induce prolonged translational pausing and ribosomal dissociation (Wen et al., 2008), thereby downregulating gene expression. Moreover, loss of exon-1 would remove diphenylalanine motifs crucial for transport of CFTR from the Golgi apparatus to the plasma membrane (Mouchel et al., 2003).

Alternative 5′-exons may also introduce a series of upstream open reading frames (uORFs) (Davies et al., 2004; Calvo et al., 2009; Barbosa et al., 2013). These elements are proximal to consensus nucleotides surrounding the initiation codon, i.e. Kozak sequence, which influence the rate of AUG recognition by ribosomes. In the 5′-UTR of native CFTR transcripts, the presence of an uORF and RNA secondary structure reduce translation initiation and mRNA stability (Lukowski et al., 2015). The Kozak context of the uORF is suboptimal with zero out of two critical nucleotides encoded, providing an infrequently recognized start codon. In addition, an RNA hairpin inhibits scanning ribosomes from potentially initiating (or re-initiating) translation at downstream AUG codons. This RNA secondary structure contributes to ribosomal stalling and dissociation as well, thereby diminishing mRNA half-life.

### CFTR sequence elements that influence splicing

Polypyrimidine sequences located near splice branch/acceptor sites impact RNA primary sequence and secondary structure. For example, thymidine-guanine (TG) repeats and polythymidine (poly-T) tract immediately adjacent to exon-10 (Figure 2) have been shown to influence alternative splicing (Chu et al., 1993; Pagani et al., 2000; Hefferon et al., 2004). An inverse relationship exists between the length of the polypyrimidine segment and subsequent mRNA abundance–e.g. an extended TG repeat sequence and poly-T tract confer enhanced exon-10 skipping, reduced CFTR transcript expression, and diminished channel activity (Chu et al., 1993; Groman et al., 2004). Formation of RNA secondary structures by dinucleotide repeats can mask splicing signals in CFTR and/or generate features that stimulate (or interfere with) protein-protein, RNA-protein, or RNA-RNA interactions (Niksic et al., 1999; Pagani et al., 2000; Buratti et al., 2001). Findings have shown higher splicing efficiency of exon-10 correlates with shorter TG repeats, which undergo self-base-pairing to form RNA hairpins with low stem loop thermostability (Hefferon et al., 2004)—suggesting that the transient presence of these structures improves pre-mRNA processing.

Splicing is also influenced by the presence of pseudoexons, which are intronic sequences that exhibit characteristics of exons (e.g., 5′- and 3′-splice sites) but are absent in mature transcripts (Grellscheid and Smith, 2006). Existence of pseudoexons in mRNA is associated with disease pathology in CF and other disorders (Dhir and Buratti, 2010; Vaz-Drago et al., 2017; Petersen et al., 2022). Earlier investigations have established that pseudoexon inclusion occurs from single nucleotide variants within *CFTR* that introduce new 5′-splice sites (Chillón et al., 1995; Buratti et al., 2007). In addition to engagement of inhibitory RNA-binding proteins and/or absence of binding sites for enhancive splicing factors, RNA secondary structures have been suggested to mediate splicing efficiency in this setting (Buratti et al., 2007). Evidence indicates that unused 5′-donor sites are common in native *CFTR* introns, although they may be positioned within RNA hairpins and therefore inaccessible (Petersen et al., 2022). Experimental manipulation of the RNA stem loops can expose the normally unemployed 5′-splice sequences, leading to inclusion of pseudoexons (Dhir and Buratti, 2010). These aspects of intron processing are important considerations for gene editing-based therapeutic strategies under development for CF.

### Attributes of the CFTR 3′-UTR

The 3′-UTR consists of non-coding mRNA between the cognate stop codon and poly-adenine tail. This sequence is best known for mediating mRNA localization, nuclear export, half-life, and translation initiation (Mignone et al., 2002; Matoulkova et al., 2012; Mayr, 2019). Alternative splicing or polyadenylation sites may produce altered 3′-UTR sequences that also affect the 3′-coding region (if the stop codon is involved) and/or regulatory features (Mayr, 2019). In the context of CFTR, adenine (A)- and uracil (U)-rich sequences have been established as strong contributors to mRNA stability (Mignone et al., 2002). CFTR possesses three AUUUA motifs, in addition to many U- and cytosine (C)-repeats (Zielenski et al., 1991). The AUUUA and poly-U tracts deplete mRNA half-life through formation of destabilizing ribonucleoprotein complexes, whereas the C-repeats putatively enhance mRNA stability through interactions with poly-C-binding proteins (Baudouin-Legros et al., 2005).

Negative post-transcriptional regulation of CFTR also occurs in cell type-specific manner through 3′-UTR interactions with microRNAs (miRNAs) (Figure 2). These molecules are single-stranded, non-coding RNAs approximately 19–25 nucleotides in length. Several miRNAs have been observed to directly bind the CFTR 3′-UTR, suppress protein production, and contribute to manifestations of CF lung disease (Megiorni et al., 2011; Ramachandran et al., 2013; Viart et al., 2015). Depletion of CFTR abundance mediated by miRNAs is further exacerbated by exposure to bacterial infection or proinflammatory cytokines, which augment expression of miRNAs that correspondingly inhibit CFTR transepithelial ion transport (Ramachandran et al., 2013).

An emerging area of CF therapeutics development involves anti-miRNA approaches. For example, miRNA-binding blocker oligonucleotides (MBBOs) are single-stranded RNA sequences engineered to mask miRNA binding sites. Recently described MBBOs employ locked nucleic acids, which are high-affinity RNA analogues that possess a ribose ring connected by a methylene bridge between the 2′-oxygen and 4′-carbon atoms (Viart et al., 2015). This modification “locks” the ribose ring in an ideal conformation for base pairing and enhances thermal stability of the MBBO-RNA hybrid. MBBOs designed to impair miRNA binding to the CFTR 3′-UTR have been shown to enhance mRNA abundance, protein maturation, and channel activity (Viart et al., 2015).

Additional miRNA-targeting technologies include peptide nucleic acids (PNAs), which are DNA analogues with a pseudo-peptide backbone composed of N-(2-aminoethyl) glycine units (Gambari et al., 2020). PNAs bind with strong affinity to complementary sequences of DNA or RNA, although uptake is limited in eukaryotic cells. The latest scientific reports indicate that PNA intercalation through the plasma membrane can be increased by loading the PNAs onto lipid-polymer nanoparticles (McNeer et al., 2015; Comegna et al., 2021; Piotrowski-Daspit et al., 2022) or linking poly-arginine tails to the PNAs (Papi et al., 2022). To date, numerous PNAs have been shown to inhibit target miRNA binding sites and augment CFTR mRNA and protein expression (McNeer et al., 2015; Zarrilli et al., 2017; Sultan et al., 2020; Fabbri et al., 2021; Piotrowski-Daspit et al., 2022). Off-target effects are an important consideration of anti-miRNA based interventions, as single miRNA species may recognize complementary sequences across multiple genes. Therefore, technologies such as PNAs or MBBOs must be carefully optimized for sequence context to mask miRNA target(s) within a specific gene of interest.

### Contributions of synonymous single nucleotide polymorphisms (SNPs)

Synonymous (or “silent”) SNPs are prevalent variations found throughout the human genome, in which a single nucleotide identity is changed without altering the encoded amino acid. While synonymous SNPs have been traditionally viewed as benign during protein biogenesis, emerging evidence indicates these polymorphisms impact polypeptide folding and sensitivity to small molecule therapies in a variety of disease states (KomarGenetics, 2007; Sauna et al., 2007; Hunt et al., 2009). Advancements in the CF field have further elucidated the impact of silent SNPs on CFTR splicing, mRNA utilization, protein maturation, and channel function.

The most commonly reported *CFTR* variant, c.1521_1523delCTT (*p*.Phe508del), is caused by deletion of cytosine in the third position of isoleucine-507 (ATC), as well as the first two thymidines present in phenylalanine-508 (TTT) (Figure 2). As a result, Phe508 is lost, and a synonymous SNP is introduced into Ile507 (ATC>ATT). Additional consequences include the appearance of two enlarged, single-stranded loops in CFTR transcripts (Bartoszewski et al., 2010). These secondary structures are predicted to cause mRNA misfolding, decreased ribosome velocity, and diminished co-translational assembly of *p*.Phe508del protein. Newer evidence indicates both ATC and ATT are decoded by similar low-abundance tRNAs (Polte et al., 2019), suggesting the proposed reduction in local ribosome speed would be independent of tRNA availability.

When the mutated Ile507-ATT codon is reverted back to ATC, the silent substitution yields native-like mRNA structure, augments *p*.Phe508del transcript levels, and amplifies protein expression of both the immature (“band B”) and mature (“band C″) glycoforms (Bartoszewski et al., 2010; Lazrak et al., 2013). This synonymous SNP would also confer a silent codon change in Phe508 (TTT>TTC), which has been documented in humans (rs1400451895) (Karczewski et al., 2020). *In vitro*, Ile507-ATC renders *p*.Phe508del more sensitive to correction by clinically approved CFTR modulators (Bali et al., 2016), indicating that synonymous SNPs may influence pharmacologic responsiveness.

Recent investigations demonstrate that additional silent SNPs induce alterations to CFTR mRNA expression, protein topology, and transepithelial ion transport (Figure 2). For example, the following three synonymous SNPs in *CFTR* exhibit significantly decreased transcript abundance: c.2280G>A (*p*.Thr760=), c.3339T>C (*p*.Ser1113=), c.3870A>G (*p*.Pro1290=) (Kirchner et al., 2017). The synonymous SNP, c.1584G>A (*p*.Glu528=), elicits retention of intron-11 and skipping of exon-11, albeit at a low frequency (Bampi et al., 2020). A prevalent silent SNP, c.2562T>G (*p*.Thr854=), reduces local ribosome speed through a mechanism dependent on tRNA abundance. This codon substitution diminishes CFTR protein stability and channel conductance (Kirchner et al., 2017). Moreover, c.2562T>G has been shown to epistatically modulate functional expression of certain rare CFTR variants. When c.2562T>G is present *in cis*, this synonymous SNP produces subtle structural rearrangements that counteract destabilizing effects of CFTR missense alleles such as *p*.Gly551Asp, *p*.Asp579Gly, and *p*.Asp614Gly (Rauscher et al., 2021). Together, findings underscore the impact that silent SNPs contribute to CFTR mRNA utilization and protein folding trajectory. Such effects are likely to influence the spectrum of disease symptoms, and may help predict therapeutic response in precision theratyping studies.

### Quality control of CFTR transcripts

Eukaryotic cells perform co-translational mRNA surveillance as a means to sustain expression of properly synthesized transcripts, as well as degrade aberrant mRNAs and recycle associated ribosomes. Three mRNA decay pathways have been extensively described: non-stop decay (NSD), no-go decay (NGD), and nonsense-mediated decay (NMD) (Shoemaker and Green, 2012; Simms et al., 2017; Embree et al., 2022). The NSD complex facilitates RNA cleavage by detaching ribosomes that become jammed at the 3′-end of transcripts lacking a native termination codon. NGD factors recognize and detach ribosomes that become stalled on transcripts bearing nucleotide modifications, secondary structures, and/or other features that interfere with efficient ribosomal decoding. NMD machinery monitors ribosomes undergoing translation termination and eliminates mRNAs harboring premature stop codons (i.e. “nonsense” alleles).

To our knowledge, NSD and NGD remain unexplored in the context of CFTR processing. These pathways still bear relevance, however, as both NSD and NGD are triggered by stalled ribosomes. CFTR is predicted to harbor ribosomal queues at stall sites as a result of elevated initiation rates (Matsumura et al., 2011; Park and Subramaniam, 2019), which may be alleviated by suppressing certain components of translation machinery. The NGD complex binds to the 60S P stalk protein, uL11 (RPL12), to facilitate ribosomal subunit disassembly (Becker et al., 2011). Approximately 50% reduction in uL11 expression attenuates translation initiation and elongation (Oliver et al., 2019). Findings also indicate uL11 depletion enhances protein maturation/stability and transepithelial ion transport exhibited by *p*.Phe508del and other CFTR missense variants (Veit et al., 2016; Oliver et al., 2019). Interestingly, siRNA-mediated knockdown of eukaryotic initiation factor 3a also improves *p*.Phe508del protein trafficking and function (Hutt et al., 2018). Tuning translation initiation by partially silencing uL11 or eukaryotic initiation factor 3a could mitigate NSD- and/or NGD-stimulated downregulation of CFTR expression conferred by stalled ribosomes.

Conversely, NMD has been experimentally verified to influence transcript abundance of numerous refractory CFTR variants (Oren et al., 2017; Sharma et al., 2018; Clarke et al., 2021). NMD is initiated by recognition of features such as long 3′-UTRs, uORFs, and/or exon-exon junction complexes downstream of stop codons [reviewed in (Yi et al., 2021)]. In humans, terminating ribosomes are distinguished by conserved up-frameshift (UPF) proteins: UPF1, UPF2, and UPF3B. UPF1 engages the mRNA and ribosome, after which UPF2 binds and triggers ATPase and helicase activity of UPF1. UPF3B stimulates UPF2-mediated activation of UPF1 and links UPF2 to exon-exon junction complexes. Transcripts become targeted for degradation in response to UPF1 phosphorylation by the serine/threonine protein kinase complex, SMG1-SMG8-SMG9. Phosphorylated UPF1 activates endonucleolytic cleavage of mRNA by SMG6, followed by deadenylation, decapping, and exonucleolytic cleavage by SMG5, SMG7, and Xrn1, respectively. Molecular agents designed to target branch-specific NMD factors are actively being pursued in preclinical testing, as a means to address defects in CFTR mRNA synthesis and stability (Table 1).

**TABLE 1:** Therapeutic strategies presently employed for CF. Clinically approved agents are emphasized, as well as interventions undergoing clinical trials or advanced preclinical testing. Abbreviations: miRNA-binding blocker oligonucleotide (MBBO); peptide nucleic acid (PNA); antisense oligonucleotide (ASO); upstream open reading frame (uORF); translation suppression element (TSE); premature termination codon (PTC); nonsense-mediated decay (NMD); anticodon edited (ACE) transfer RNA (tRNA); exonic splicing enhancer (ESE); lipid nanoparticle (LNP); adeno-associated virus (AAV).

Therapeutic strategy		Sponsor	Intervention(s)	Clinical stage	References and/or clinicaltrials.gov identifiers
Gating and/or conductance variants					
Small molecules	Potentiator	Vertex Pharmaceuticals	Ivacaftor (VX-770)	Approved	KALYDECO (2020)
	Potentiator	Vertex Pharmaceuticals	Deuterated-Ivacaftor (VX-561)	Phase II	NCT03911713
Processing, trafficking, and/or expression variants					
Small molecules	1 Corrector +1 Potentiator	Vertex Pharmaceuticals	Lumacaftor (VX-809) + Ivacaftor	Approved	ORKAMBI (2019)
	1 Corrector +1 Potentiator	Vertex Pharmaceuticals	Tezacaftor (VX-661) + Ivacaftor	Approved	SYMDEKO (2020)
	2 Correctors +1 Potentiator	Vertex Pharmaceuticals	Elexacaftor (VX-445) + Tezacaftor + Ivacaftor	Approved	TRIKAFTA (2021)
	2 Correctors +1 Potentiator	Vertex Pharmaceuticals	VX-121 + Tezacaftor + Deuterated-Ivacaftor	Phase III	NCT05422222
					NCT05444257
					NCT05033080
					NCT05076149
	2 Correctors +1 Potentiator	Abbvie	ABBV-2222 + ABBV-3067 + ABBV-576	Phase II	NCT03969888
	Amplifier	Proteostasis Therapeutics	Nesolicaftor (PTI-428)	Reached Phase II trials before testing ended due to merger with Yumanity Therapeutics	NCT03591094
					NCT03500263 (PR Newswire, 2020)
MBBOs	miRNA blocker	Academic investigators	MBBO-1, MBBO-2, MBBO-3, MBBO-4	Preclinical	Viart et al. (2015)
PNAs	miRNA blocker	Academic investigators	ɣPNA/DNA nanoparticles; PNA-a145, PNA-a101	Preclinical	McNeer et al. (2015), Zarrilli et al. (2017), Sultan et al. (2020), Fabbri et al. (2021), Papi et al. (2022), Piotrowski-Daspit et al. (2022)
ASOs	Steric blockers of 5′-UTR uORF or TSE stem loop	Ionis Pharmaceuticals	uORF-ASO, TSE-ASO	Preclinical	Sasaki et al. (2019)
Nonsense variants					
Small molecules	PTC read-through	Eloxx Pharmaceuticals	Exaluren (ELX-02)	Phase II	NCT04135495
					NCT04126473
	PTC read-through	PTC Therapeutics	Ataluren (PTC-124)	Reached Phase III and “N-of-1” Phase IV trials before testing ended due to failure of meeting clinical endpoints	NCT03256968
					NCT03256799
					NCT00803205
					NCT01140451
					NCT02139306
	PTC read-through	Southern Research Institute	SRI-41315	Preclinical	Ng et al. (2021), Sharma et al. (2021)
	NMD inhibition	Cystic Fibrosis Foundation	SMG1i	Preclinical	Gopalsamy et al. (2012), Laselva et al. (2020), Venturini et al. (2021), de Poel et al. (2022)
	NMD inhibition	Academic investigators	CC-90009	Preclinical	Baradaran-Heravi et al. (2021), Nishiguchi et al. (2021), Lee et al. (2022)
	NMD inhibition	Academic investigators	SJ6986	Preclinical	Baradaran-Heravi et al. (2021), Nishiguchi et al. (2021), Lee et al. (2022)
ASOs	NMD inhibition	Ionis Pharmaceuticals	SMG1-ASO, SMG6-ASO	Preclinical	Keenan et al. (2019), Sanderlin et al. (2022)
tRNA suppressors	PTC read-through	Academic investigators	ACE-tRNAs	Preclinical	Lueck et al. (2019), Porter et al. (2021), Ko et al. (2022)
Splice variants					
ASOs	Splice-switching	SpliSense	SPL84	Phase I/II	Oren et al. (2021), Oren et al. (2022), PR Newswire (2022)
	Splice-switching	Academic investigators	ASO-23A, ASO-23B; ESE-1, ESE-2, ESE-3	Preclinical	Michaels et al. (2020), Kim et al. (2022), Michaels et al. (2022)
Variant-agnostic approaches					
LNP vehicle	mRNA delivery	Arcturus Therapeutics	LUNAR^®^ platform (ARCT-032)	Phase I	NCT05712538
	mRNA delivery	Vertex Pharmaceuticals	VX-522	Phase I	NCT05668741
	mRNA delivery	ReCode Therapeutics	SORT-LNP^TM^ platform	Preclinical	BusinessWire (2023)
AAV vector	Gene delivery	4D Molecular Therapeutics	4D-710	Phase I	NCT05248230

### RNA-directed therapeutic strategies

Multiple subtypes of *CFTR* variants–such as frameshifts, splicing defects, and nonsense codons–exhibit susceptibility to premature mRNA decay and protein degradation. These variants are largely insensitive to (and ineligible for) clinically approved CFTR modulators, thus representing a significant unmet medical need. Recent emphasis has been placed on development of gene transfer, gene editing, and mRNA therapies (reviewed in (Da Silva Sanchez et al., 2020; Egan, 2021; Harrison, 2022; Sui et al., 2022)). Here, we briefly summarize small molecule and short nucleotide-based approaches that directly interact with translation machinery to circumvent cellular disposal of aberrant CFTR transcripts and associated polypeptides (Table 1).

Compounds that stabilize CFTR mRNA, and therefore augment both immature and mature CFTR protein, are termed “amplifiers” (Molinski et al., 2017). Certain small molecules in this class increase CFTR transcript abundance and translation efficiency through enhanced association with polysomes and poly (rC)-binding protein 1 (PCBP1) (Giuliano et al., 2018; Dukovski et al., 2020). These amplifiers require a PCBP1 consensus element within the CFTR ORF to elicit effects on translation. Due to a distinct mechanism of action, many amplifiers demonstrate additivity or synergy with clinically approved CFTR modulators (Molinski et al., 2017; Smith et al., 2021; Bengtson et al., 2022). When combined with the highly effective modulator therapy, elexacaftor-tezacaftor-ivacaftor, the amplifier PTI-428 (nesolicaftor) augments mRNA half-life and protein function of the *p*.Phe508del CFTR variant (Bengtson et al., 2022). Moreover, PTI-428 overcomes the negative effects of miRNA expression on CFTR mRNA abundance, and reduces cytokine production in the setting of acute inflammation (Bengtson et al., 2022).

Robust attention has also been devoted towards engineering small molecules that restore full-length protein expression of CFTR nonsense variants. Translation is arrested when a ribosome encounters any of the three termination codons (UAA, UAG, UGA), which are recognized by eukaryotic release factor 1 (eRF1). Following GTP hydrolysis by eRF3a, the nascent peptide is released and ribosomal subunits are disassembled (Hellen, 2018). Compounds that specifically deplete eRF1 or eRF3a enhance full-length protein production and channel activity of CFTR premature termination codons (Sharma et al., 2021; Lee et al., 2022). Additional small molecules have been designed to reduce ribosome fidelity through a variety of mechanisms and confer read-through of CFTR nonsense alleles (Du et al., 2008; Crawford et al., 2021), although none of these pharmacologic agents have been clinically approved (Table 1).

Suppressor tRNAs have emerged as a promising new method for overcoming CFTR nonsense alleles. For example, anticodon engineered transfer RNAs (ACE-tRNAs) possess altered anticodons that pair to termination codons (Porter et al., 2021). ACE-tRNAs are stabilized by post-transcriptional modifications and charged by endogenous aminoacyl-tRNA synthetases. Together, these alterations allow ACE-tRNAs to read-through CFTR premature stops, effectively rescuing channel function of certain CFTR nonsense variants to levels predictive of clinical benefit (Lueck et al., 2019; Ko et al., 2022). Additional species of suppressor tRNAs have been engineered with pseudouridine (Ψ) modifications in the anticodon stem loop, which confer stop codon read-through *via* incorporation of near-cognate amino acids (Albers et al., 2021). Although to date, this approach has only proven successful in bacteria.

Antisense oligonucleotides (ASOs) are presently under investigation as a therapeutic strategy for NMD inhibition and regulation of alternative splicing (Table 1). ASOs are short (∼15–30 nucleotides), single-stranded DNA, RNA, or DNA:RNA analogs that bind and functionally alter complementary RNA sequences. Chemical modifications to ASOs can either: (1) sterically impair RNA-binding proteins, nucleic acids, or other factors involved in RNA utilization (Khorkova and Wahlestedt, 2017); or (2) recruit RNase-H to facilitate degradation of the RNA target (Crooke, 2017). ASOs employing steric hindrance have been clinically approved to treat diseases apart from CF, and have been well-tolerated in thousands of human subjects using a number of administration routes (Mahfouz et al., 2020; Singh et al., 2020; Akoumianakis et al., 2021). These ASOs do not require a delivery vehicle, thereby reducing risk of innate or adaptive immune responses in patients receiving repeat doses.

In the setting of CF, preclinical investigations indicate ASO-dependent silencing of SMG1 or SMG6 significantly increases transcript expression, protein trafficking, and transepithelial ion transport of CFTR nonsense variants (Keenan et al., 2019; Sanderlin et al., 2022). ASOs that sterically block uORFs in the 5′-UTR have also been shown to augment transcription and function of CFTR (Sasaki et al., 2019). Other ASOs have been developed as splice-switching oligonucleotides that overcome cryptic splice site activation. For example, the *CFTR* variant c.3718–2477C>T (3849+10kbC>T) generates a new 5′-splice site that causes incorporation of a cryptic exon bearing a nonsense codon (Highsmith et al., 1994). ASO-mediated inhibition of the alternative splice site prevents inclusion of the cryptic exon and restores function to 3849+10kbC>T (Michaels et al., 2020; Oren et al., 2021). This approach is presently undergoing clinical testing (PR Newswire, 2022).

Additional splice-switching ASOs facilitate exon removal. ASO-dependent skipping of exon-23 rescues functional expression of CFTR premature termination codons such as *p*.Trp1282Ter (Kim et al., 2022; Michaels et al., 2022; Oren et al., 2022). These ASOs preserve the CFTR reading frame while conferring enhanced mRNA stabilization and channel activity. Because C-terminal residues important for post-translational processing and channel gating would be retained, this strategy is being pursued for splicing and nonsense variants that occur within exon-23 and other in-frame *CFTR* exons (e.g. exon-9) (Martinovich et al., 2022). Minimizing exposure of NMD suppression and/or splice-switching to localized area(s) of delivery remains a prominent consideration.

## Discussion

Advancements in CFTR modulator treatments have revolutionized approaches to personalized medicine for people living with CF. However, effective therapeutic strategies are still needed to address underlying genetic defects for patients with presently untreatable forms of the disease. Sequence context plays an important role during CFTR processing, although contributing mechanisms remain poorly understood. Nucleotide identity, mRNA secondary structures, and additional features surrounding the variant–including proximity to the 5′- or 3′-end of *CFTR*–are likely to influence capacity for functional restoration. Tuning ribosome fidelity, translational velocity, and/or mRNA surveillance are viable methods for improving mutant CFTR biosynthesis. Central to the success of such approaches is CFTR mRNA quality. Future studies will be required to elucidate the most ideal sequence and/or modifications necessary for optimal transcript abundance, stability, and utilization.

## References

[B1] Akoumianakis I. Zvintzou E. Kypreos K. Filippatos T. D. (2021). ANGPTL3 and apolipoprotein C-III as novel lipid-lowering targets. Curr. Atheroscler. Rep. 23 (5), 20. 10.1007/s11883-021-00914-7 33694000

[B2] Albers S. Beckert B. Matthies M. C. Mandava C. S. Schuster R. Seuring C. (2021). Repurposing tRNAs for nonsense suppression. Nat. Commun. 12 (1), 3850. 10.1038/s41467-021-24076-x 34158503 PMC8219837

[B3] Bali V. Lazrak A. Guroji P. Fu L. Matalon S. Bebok Z. (2016). A synonymous codon change alters the drug sensitivity of ΔF508 cystic fibrosis transmembrane conductance regulator. Faseb J. 30 (1), 201–213. 10.1096/fj.15-273714 26336913 PMC4684529

[B4] Bampi G. B. Ramalho A. S. Santos L. A. Wagner J. Dupont L. Cuppens H. (2020). The effect of synonymous single-nucleotide polymorphisms on an atypical cystic fibrosis clinical presentation. Life (Basel) 11, 14. 10.3390/life11010014 33375403 PMC7824434

[B5] Baradaran-Heravi A. Balgi A. D. Hosseini-Farahabadi S. Choi K. Has C. Roberge M. (2021). Effect of small molecule eRF3 degraders on premature termination codon readthrough. Nucleic Acids Res. 49 (7), 3692–3708. 10.1093/nar/gkab194 33764477 PMC8053119

[B6] Barbosa C. Peixeiro I. Romão L. (2013). Gene expression regulation by upstream open reading frames and human disease. PLoS Genet. 9 (8), e1003529. 10.1371/journal.pgen.1003529 23950723 PMC3738444

[B7] Bartoszewski R. A. Jablonsky M. Bartoszewska S. Stevenson L. Dai Q. Kappes J. (2010). A synonymous single nucleotide polymorphism in DeltaF508 CFTR alters the secondary structure of the mRNA and the expression of the mutant protein. J. Biol. Chem. 285 (37), 28741–28748. 10.1074/jbc.M110.154575 20628052 PMC2937902

[B8] Baudouin-Legros M. Hinzpeter A. Jaulmes A. Brouillard F. Costes B. Fanen P. (2005). Cell-specific posttranscriptional regulation of CFTR gene expression via influence of MAPK cascades on 3'UTR part of transcripts. Am. J. Physiol. Cell. Physiol. 289 (5), C1240–C1250. 10.1152/ajpcell.00595.2004 15944206

[B9] Becker T. Armache J. P. Jarasch A. Anger A. M. Villa E. Sieber H. (2011). Structure of the no-go mRNA decay complex Dom34-Hbs1 bound to a stalled 80S ribosome. Nat. Struct. Mol. Biol. 18 (6), 715–720. 10.1038/nsmb.2057 21623367

[B10] Ben-Dov C. Hartmann B. Lundgren J. Valcárcel J. (2008). Genome-wide analysis of alternative pre-mRNA splicing. J. Biol. Chem. 283 (3), 1229–1233. 10.1074/jbc.R700033200 18024428

[B11] Bengtson C. Silswal N. Baumlin N. Yoshida M. Dennis J. Yerrathota S. (2022). The CFTR Amplifier Nesolicaftor Rescues TGF-β1 Inhibition of Modulator-Corrected F508del CFTR Function. Int. J. Mol. Sci. 23, 810956. 10.3390/ijms231810956 PMC9504033 36142862

[B12] Buratti E. Dörk T. Zuccato E. Pagani F. Romano M. Baralle F. E. (2001). Nuclear factor TDP-43 and SR proteins promote *in vitro* and *in vivo* CFTR exon 9 skipping. Embo J. 20 (7), 1774–1784. 10.1093/emboj/20.7.1774 11285240 PMC145463

[B13] Buratti E. Dhir A. Lewandowska M. A. Baralle F. E. (2007). RNA structure is a key regulatory element in pathological ATM and CFTR pseudoexon inclusion events. Nucleic Acids Res. 35 (13), 4369–4383. 10.1093/nar/gkm447 17580311 PMC1935003

[B14] BusinessWire (2023). ReCode Therapeutics announces strategic investment from the Cystic Fibrosis Foundation to accelerate development of novel mRNA therapy [press release]. Menlo Park, CA: BusinessWire. Available from: https://www.businesswire.com/news/home/20230110005385/en/ .

[B15] Calvo S. E. Pagliarini D. J. Mootha V. K. (2009). Upstream open reading frames cause widespread reduction of protein expression and are polymorphic among humans. Proc. Natl. Acad. Sci. U. S. A. 106 (18), 7507–7512. 10.1073/pnas.0810916106 19372376 PMC2669787

[B16] Chillón M. Dörk T. Casals T. Giménez J. Fonknechten N. Will K. (1995). A novel donor splice site in intron 11 of the CFTR gene, created by mutation 1811+1.6kbA-->G, produces a new exon: High frequency in Spanish cystic fibrosis chromosomes and association with severe phenotype. Am. J. Hum. Genet. 56 (3), 623–629. 7534040 PMC1801150

[B17] Chou J. L. Rozmahel R. Tsui L. C. (1991). Characterization of the promoter region of the cystic fibrosis transmembrane conductance regulator gene. J. Biol. Chem. 266 (36), 24471–24476. 10.1016/s0021-9258(18)54252-6 1722205

[B18] Chu C. S. Trapnell B. C. Curristin S. Cutting G. R. Crystal R. G. (1993). Genetic basis of variable exon 9 skipping in cystic fibrosis transmembrane conductance regulator mRNA. Nat. Genet. 3 (2), 151–156. 10.1038/ng0293-151 7684646

[B19] Clarke L. A. Luz V. C. C. Targowski S. Ramalho S. S. Farinha C. M. Amaral M. D. (2021). Integrity and stability of PTC bearing CFTR mRNA and relevance to future modulator therapies in cystic fibrosis. Genes. (Basel) 12, 1810. 10.3390/genes12111810 34828417 PMC8621375

[B20] Collins F. S. (1992). Cystic fibrosis: Molecular biology and therapeutic implications. Science 256 (5058), 774–779. 10.1126/science.1375392 1375392

[B21] Comegna M. Conte G. Falanga A. P. Marzano M. Cernera G. Di Lullo A. M. (2021). Assisting PNA transport through cystic fibrosis human airway epithelia with biodegradable hybrid lipid-polymer nanoparticles. Sci. Rep. 11 (1), 6393. 10.1038/s41598-021-85549-z 33737583 PMC7973768

[B22] Cottrill K. A. Farinha C. M. McCarty N. A. (2020). The bidirectional relationship between CFTR and lipids. Commun. Biol. 3 (1), 179. 10.1038/s42003-020-0909-1 32313074 PMC7170930

[B23] Crawford D. K. Mullenders J. Pott J. Boj S. F. Landskroner-Eiger S. Goddeeris M. M. (2021). Targeting G542X CFTR nonsense alleles with ELX-02 restores CFTR function in human-derived intestinal organoids. J. Cyst. Fibros. 20 (3), 436–442. 10.1016/j.jcf.2021.01.009 33558100

[B24] Crooke S. T. (2017). Molecular mechanisms of antisense oligonucleotides. Nucleic Acid. Ther. 27 (2), 70–77. 10.1089/nat.2016.0656 28080221 PMC5372764

[B25] Cystic Fibrosis Mutation Database (2022). The hospital for sick children. Available from: http://www.genet.sickkids.on.ca/Home.html .

[B26] Da Silva Sanchez A. Paunovska K. Cristian A. Dahlman J. E. (2020). Treating cystic fibrosis with mRNA and CRISPR. Hum. Gene Ther. 31 (17-18), 940–955. 10.1089/hum.2020.137 32799680 PMC7495921

[B27] Davies W. L. Vandenberg J. I. Sayeed R. A. Trezise A. E. (2004). Cardiac expression of the cystic fibrosis transmembrane conductance regulator involves novel exon 1 usage to produce a unique amino-terminal protein. J. Biol. Chem. 279 (16), 15877–15887. 10.1074/jbc.M313628200 14754881

[B28] De Boeck K. Amaral M. D. (2016). Progress in therapies for cystic fibrosis. Lancet Respir. Med. 4 (8), 662–674. 10.1016/S2213-2600(16)00023-0 27053340

[B29] de Poel E. Spelier S. Suen S. W. F. Kruisselbrink E. Graeber S. Y. Mall M. A. (2022). Functional restoration of CFTR nonsense mutations in intestinal organoids. J. Cyst. Fibros. 21 (2), 246–253. 10.1016/j.jcf.2021.09.020 34666947

[B30] Dhir A. Buratti E. (2010). Alternative splicing: Role of pseudoexons in human disease and potential therapeutic strategies. Febs J. 277 (4), 841–855. 10.1111/j.1742-4658.2009.07520.x 20082636

[B31] Du M. Liu X. Welch E. M. Hirawat S. Peltz S. W. Bedwell D. M. (2008). PTC124 is an orally bioavailable compound that promotes suppression of the human CFTR-G542X nonsense allele in a CF mouse model. Proc. Natl. Acad. Sci. U. S. A. 105 (6), 2064–2069. 10.1073/pnas.0711795105 18272502 PMC2538881

[B32] Dukovski D. Villella A. Bastos C. King R. Finley D. Kelly J. W. (2020). Amplifiers co-translationally enhance CFTR biosynthesis via PCBP1-mediated regulation of CFTR mRNA. J. Cyst. Fibros. 19 (5), 733–741. 10.1016/j.jcf.2020.02.006 32067958

[B33] Egan M. E. (2021). Emerging technologies for cystic fibrosis transmembrane conductance regulator restoration in all people with CF. Pediatr. Pulmonol. 56, S32–S39. 10.1002/ppul.24965 32681713 PMC8114183

[B34] Embree C. M. Abu-Alhasan R. Singh G. (2022). Features and factors that dictate if terminating ribosomes cause or counteract nonsense-mediated mRNA decay. J. Biol. Chem. 298 (11), 102592. 10.1016/j.jbc.2022.102592 36244451 PMC9661723

[B35] Ensinck M. M. Carlon M. S. (2022). One size does not fit all: The past, present and future of cystic fibrosis causal therapies. Cells 11, 1868. 10.3390/cells11121868 35740997 PMC9220995

[B36] Fabbri E. Tamanini A. Jakova T. Gasparello J. Manicardi A. Corradini R. (2021). Treatment of human airway epithelial Calu-3 cells with a peptide-nucleic acid (PNA) targeting the microRNA miR-101-3p is associated with increased expression of the cystic fibrosis Transmembrane Conductance Regulator gene. Eur. J. Med. Chem. 209, 112876. 10.1016/j.ejmech.2020.112876 33127171

[B37] Gambari R. Gasparello J. Fabbri E. Borgatti M. Tamanini A. Finotti A. (2020). Peptide nucleic acids for MicroRNA targeting. Methods Mol. Biol. 2105, 199–215. 10.1007/978-1-0716-0243-0_12 32088872

[B38] Giuliano K. A. Wachi S. Drew L. Dukovski D. Green O. Bastos C. (2018). Use of a high-throughput phenotypic screening strategy to identify amplifiers, a novel pharmacological class of small molecules that exhibit functional synergy with potentiators and correctors. SLAS Discov. 23 (2), 111–121. 10.1177/2472555217729790 28898585 PMC5784457

[B39] Gopalsamy A. Bennett E. M. Shi M. Zhang W. G. Bard J. Yu K. (2012). Identification of pyrimidine derivatives as hSMG-1 inhibitors. Bioorg Med. Chem. Lett. 22 (21), 6636–6641. 10.1016/j.bmcl.2012.08.107 23021994

[B40] Grellscheid S. N. Smith C. W. (2006). An apparent pseudo-exon acts both as an alternative exon that leads to nonsense-mediated decay and as a zero-length exon. Mol. Cell. Biol. 26 (6), 2237–2246. 10.1128/MCB.26.6.2237-2246.2006 16508000 PMC1430291

[B41] Groman J. D. Hefferon T. W. Casals T. Bassas L. Estivill X. Des Georges M. (2004). Variation in a repeat sequence determines whether a common variant of the cystic fibrosis transmembrane conductance regulator gene is pathogenic or benign. Am. J. Hum. Genet. 74 (1), 176–179. 10.1086/381001 14685937 PMC1181905

[B42] Harrison P. T. (2022). CFTR RNA- and DNA-based therapies. Curr. Opin. Pharmacol. 65, 102247. 10.1016/j.coph.2022.102247 35709547

[B43] Hefferon T. W. Groman J. D. Yurk C. E. Cutting G. R. (2004). A variable dinucleotide repeat in the CFTR gene contributes to phenotype diversity by forming RNA secondary structures that alter splicing. Proc. Natl. Acad. Sci. U. S. A. 101 (10), 3504–3509. 10.1073/pnas.0400182101 14993601 PMC373492

[B44] Hellen C. U. T. (2018). Translation termination and ribosome recycling in eukaryotes. Cold Spring Harb. Perspect. Biol. 10, a032656. 10.1101/cshperspect.a032656 29735640 PMC6169810

[B45] Highsmith W. E. Burch L. H. Zhou Z. Olsen J. C. Boat T. E. Spock A. (1994). A novel mutation in the cystic fibrosis gene in patients with pulmonary disease but normal sweat chloride concentrations. N. Engl. J. Med. 331 (15), 974–980. 10.1056/NEJM199410133311503 7521937

[B46] Hunt R. Sauna Z. E. Ambudkar S. V. Gottesman M. M. Kimchi-Sarfaty C. (2009). Silent (synonymous) SNPs: Should we care about them? Methods Mol. Biol. 578, 23–39. 10.1007/978-1-60327-411-1_2 19768585

[B47] Hutt D. M. Loguercio S. Roth D. M. Su A. I. Balch W. E. (2018). Correcting the F508del-CFTR variant by modulating eukaryotic translation initiation factor 3-mediated translation initiation. J. Biol. Chem. 293 (35), 13477–13495. 10.1074/jbc.RA118.003192 30006345 PMC6120211

[B48] KALYDECO (2020). KALYDECO [prescribing information]. Boston, MA: Vertex Pharmaceuticals Incorporated. Available from: https://www.kalydeco.com/ .

[B49] Karczewski K. J. Francioli L. C. Tiao G. Cummings B. B. Alföldi J. Wang Q. (2020). The mutational constraint spectrum quantified from variation in 141,456 humans. Nature 581 (7809), 434–443. 10.1038/s41586-020-2308-7 32461654 PMC7334197

[B50] Keenan M. M. Huang L. Jordan N. J. Wong E. Cheng Y. Valley H. C. (2019). Nonsense-mediated RNA decay pathway inhibition restores expression and function of W1282X CFTR. Am. J. Respir. Cell. Mol. Biol. 61 (3), 290–300. 10.1165/rcmb.2018-0316OC 30836009

[B51] Khorkova O. Wahlestedt C. (2017). Oligonucleotide therapies for disorders of the nervous system. Nat. Biotechnol. 35 (3), 249–263. 10.1038/nbt.3784 28244991 PMC6043900

[B52] Kim Y. J. Sivetz N. Layne J. Voss D. M. Yang L. Zhang Q. (2022). Exon-skipping antisense oligonucleotides for cystic fibrosis therapy. Proc. Natl. Acad. Sci. U. S. A. 119, e2114858118. 10.1073/pnas.2114858118 35017301 PMC8784140

[B53] Kirchner S. Cai Z. Rauscher R. Kastelic N. Anding M. Czech A. (2017). Alteration of protein function by a silent polymorphism linked to tRNA abundance. PLoS Biol. 15 (5), e2000779. 10.1371/journal.pbio.2000779 28510592 PMC5433685

[B54] Ko W. Porter J. J. Sipple M. T. Edwards K. M. Lueck J. D. (2022). Efficient suppression of endogenous CFTR nonsense mutations using anticodon-engineered transfer RNAs. Mol. Ther. Nucleic Acids 28, 685–701. 10.1016/j.omtn.2022.04.033 35664697 PMC9126842

[B55] Koh J. Sferra T. J. Collins F. S. (1993). Characterization of the cystic fibrosis transmembrane conductance regulator promoter region. Chromatin context and tissue-specificity. J. Biol. Chem. 268 (21), 15912–15921. 10.1016/s0021-9258(18)82339-0 7688000

[B56] KomarGenetics A. A. (2007). SNPs, silent but not invisible. Science 315 (5811), 466–467. 10.1126/science.1138239 17185559

[B57] Laselva O. Eckford P. D. Bartlett C. Ouyang H. Gunawardena T. N. Gonska T. (2020). Functional rescue of c.3846G>A (W1282X) in patient-derived nasal cultures achieved by inhibition of nonsense mediated decay and protein modulators with complementary mechanisms of action. J. Cyst. Fibros. 19 (5), 717–727. 10.1016/j.jcf.2019.12.001 31831337

[B58] Lazrak A. Fu L. Bali V. Bartoszewski R. Rab A. Havasi V. (2013). The silent codon change I507-ATC->ATT contributes to the severity of the ΔF508 CFTR channel dysfunction. Faseb J. 27 (11), 4630–4645. 10.1096/fj.13-227330 23907436 PMC4046180

[B59] Lee R. E. Lewis C. A. He L. Bulik-Sullivan E. C. Gallant S. C. Mascenik T. M. (2022). Small-molecule eRF3a degraders rescue CFTR nonsense mutations by promoting premature termination codon readthrough. J. Clin. Invest. 132, e154571. 10.1172/JCI154571 35900863 PMC9479597

[B60] Lueck J. D. Yoon J. S. Perales-Puchalt A. Mackey A. L. Infield D. T. Behlke M. A. (2019). Engineered transfer RNAs for suppression of premature termination codons. Nat. Commun. 10 (1), 822. 10.1038/s41467-019-08329-4 30778053 PMC6379413

[B61] Lukowski S. W. Rothnagel J. A. Trezise A. E. (2015). CFTR mRNA expression is regulated by an upstream open reading frame and RNA secondary structure in its 5' untranslated region. Hum. Mol. Genet. 24 (4), 899–912. 10.1093/hmg/ddu501 25274779

[B62] Mahfouz M. Maruyama R. Yokota T. (2020). Inotersen for the treatment of hereditary transthyretin amyloidosis. Methods Mol. Biol. 2176, 87–98. 10.1007/978-1-0716-0771-8_6 32865784

[B63] Marson F. A. L. (2018). Disease-modifying genetic factors in cystic fibrosis. Curr. Opin. Pulm. Med. 24 (3), 296–308. 10.1097/MCP.0000000000000479 29517584

[B64] Martinovich K. M. Kicic A. Stick S. M. Johnsen R. D. Fletcher S. Wilton S. D. (2022). Investigating the implications of CFTR exon skipping using a cftr exon 9 deleted mouse model. Front. Pharmacol. 13, 868863. 10.3389/fphar.2022.868863 35392567 PMC8981082

[B65] Matoulkova E. Michalova E. Vojtesek B. Hrstka R. (2012). The role of the 3' untranslated region in post-transcriptional regulation of protein expression in mammalian cells. RNA Biol. 9 (5), 563–576. 10.4161/rna.20231 22614827

[B66] Matsumura Y. Rooney L. Skach W. R. (2011). *In vitro* methods for CFTR biogenesis. Methods Mol. Biol. 741, 233–253. 10.1007/978-1-61779-117-8_16 21594789

[B67] Mayr C. (2019). What are 3' UTRs doing? Cold Spring Harb. Perspect. Biol. 11, a034728. 10.1101/cshperspect.a034728 30181377 PMC6771366

[B68] McGarry M. E. McColley S. A. (2021). Cystic fibrosis patients of minority race and ethnicity less likely eligible for CFTR modulators based on CFTR genotype. Pediatr. Pulmonol. 56 (6), 1496–1503. 10.1002/ppul.25285 33470563 PMC8137541

[B69] McNeer N. A. Anandalingam K. Fields R. J. Caputo C. Kopic S. Gupta A. (2015). Nanoparticles that deliver triplex-forming peptide nucleic acid molecules correct F508del CFTR in airway epithelium. Nat. Commun. 6, 6952. 10.1038/ncomms7952 25914116 PMC4480796

[B70] Megiorni F. Cialfi S. Dominici C. Quattrucci S. Pizzuti A. (2011). Synergistic post-transcriptional regulation of the Cystic Fibrosis Transmembrane conductance Regulator (CFTR) by miR-101 and miR-494 specific binding. PLoS One 6 (10), e26601. 10.1371/journal.pone.0026601 22028919 PMC3197680

[B71] Michaels W. E. Bridges R. J. Hastings M. L. (2020). Antisense oligonucleotide-mediated correction of CFTR splicing improves chloride secretion in cystic fibrosis patient-derived bronchial epithelial cells. Nucleic Acids Res. 48 (13), 7454–7467. 10.1093/nar/gkaa490 32520327 PMC7367209

[B72] Michaels W. E. Pena-Rasgado C. Kotaria R. Bridges R. J. Hastings M. L. (2022). Open reading frame correction using splice-switching antisense oligonucleotides for the treatment of cystic fibrosis. Proc. Natl. Acad. Sci. U. S. A. 119, e2114886119. 10.1073/pnas.2114886119 35017302 PMC8784102

[B73] Mignone F. Gissi C. Liuni S. Pesole G. (2002). Untranslated regions of mRNAs. Genome Biol. 3, Reviews0004. 10.1186/gb-2002-3-3-reviews0004 11897027 PMC139023

[B74] Molinski S. V. Ahmadi S. Ip W. Ouyang H. Villella A. Miller J. P. (2017). Orkambi® and amplifier co-therapy improves function from a rare CFTR mutation in gene-edited cells and patient tissue. EMBO Mol. Med. 9 (9), 1224–1243. 10.15252/emmm.201607137 28667089 PMC5582412

[B75] Mouchel N. Broackes-Carter F. Harris A. (2003). Alternative 5' exons of the CFTR gene show developmental regulation. Hum. Mol. Genet. 12 (7), 759–769. 10.1093/hmg/ddg079 12651871

[B76] Ng M. Y. Li H. Ghelfi M. D. Goldman Y. E. Cooperman B. S. (2021). Ataluren and aminoglycosides stimulate read-through of nonsense codons by orthogonal mechanisms. Proc. Natl. Acad. Sci. U. S. A. 118, e2020599118. 10.1073/pnas.2020599118 33414181 PMC7812769

[B77] Niksic M. Romano M. Buratti E. Pagani F. Baralle F. E. (1999). Functional analysis of cis-acting elements regulating the alternative splicing of human CFTR exon 9. Hum. Mol. Genet. 8 (13), 2339–2349. 10.1093/hmg/8.13.2339 10556281

[B78] Nishiguchi G. Keramatnia F. Min J. Chang Y. Jonchere B. Das S. (2021). Identification of potent, selective, and orally bioavailable small-molecule GSPT1/2 degraders from a focused library of cereblon modulators. J. Med. Chem. 64 (11), 7296–7311. 10.1021/acs.jmedchem.0c01313 34042448 PMC8201443

[B79] Oliver K. E. Han S. T. Sorscher E. J. Cutting G. R. (2017). Transformative therapies for rare CFTR missense alleles. Curr. Opin. Pharmacol. 34, 76–82. 10.1016/j.coph.2017.09.018 29032041 PMC5723219

[B80] Oliver K. E. Rauscher R. Mijnders M. Wang W. Wolpert M. J. Maya J. (2019). Slowing ribosome velocity restores folding and function of mutant CFTR. J. Clin. Invest. 129 (12), 5236–5253. 10.1172/JCI124282 31657788 PMC6877332

[B81] Oren Y. S. Pranke I. M. Kerem B. Sermet-Gaudelus I. (2017). The suppression of premature termination codons and the repair of splicing mutations in CFTR. Curr. Opin. Pharmacol. 34, 125–131. 10.1016/j.coph.2017.09.017 29128743

[B82] Oren Y. S. Irony-Tur Sinai M. Golec A. Barchad-Avitzur O. Mutyam V. Li Y. (2021). Antisense oligonucleotide-based drug development for Cystic Fibrosis patients carrying the 3849+10 kb C-to-T splicing mutation. J. Cyst. Fibros. 20 (5), 865–875. 10.1016/j.jcf.2021.06.003 34226157 PMC8464507

[B83] Oren Y. S. Avizur-Barchad O. Ozeri-Galai E. Elgrabli R. Schirelman M. R. Blinder T. (2022). Antisense oligonucleotide splicing modulation as a novel Cystic Fibrosis therapeutic approach for the W1282X nonsense mutation. J. Cyst. Fibros. 21, 630–636. 10.1016/j.jcf.2021.12.012 34972649

[B84] ORKAMBI (2019). ORKAMBI [prescribing information]. Boston, MA: Vertex Pharmaceuticals Incorporated. Available from: https://www.orkambi.com/ .

[B85] Pagani F. Buratti E. Stuani C. Romano M. Zuccato E. Niksic M. (2000). Splicing factors induce cystic fibrosis transmembrane regulator exon 9 skipping through a nonevolutionary conserved intronic element. J. Biol. Chem. 275 (28), 21041–21047. 10.1074/jbc.M910165199 10766763

[B86] Papi C. Gasparello J. Zurlo M. Manicardi A. Corradini R. Cabrini G. (2022). Combined treatment of bronchial epithelial calu-3 cells with peptide nucleic acids targeting miR-145-5p and miR-101-3p: Synergistic enhancement of the expression of the cystic fibrosis transmembrane conductance regulator (CFTR) gene. Int. J. Mol. Sci. 23, 9348. 10.3390/ijms23169348 36012615 PMC9409490

[B87] Park H. Subramaniam A. R. (2019). Inverted translational control of eukaryotic gene expression by ribosome collisions. PLoS Biol. 17 (9), e3000396. 10.1371/journal.pbio.3000396 31532761 PMC6750593

[B88] Petersen U. S. S. Doktor T. K. Andresen B. S. (2022). Pseudoexon activation in disease by non-splice site deep intronic sequence variation - wild type pseudoexons constitute high-risk sites in the human genome. Hum. Mutat. 43 (2), 103–127. 10.1002/humu.24306 34837434

[B89] Piotrowski-Daspit A. S. Barone C. Lin C. Y. Deng Y. Wu D. Binns T. C. (2022). *In vivo* correction of cystic fibrosis mediated by PNA nanoparticles. Sci. Adv. 8, eabo0522. 10.1126/sciadv.abo0522 36197984 PMC9534507

[B90] Polte C. Wedemeyer D. Oliver K. E. Wagner J. Bijvelds M. J. C. Mahoney J. (2019). Assessing cell-specific effects of genetic variations using tRNA microarrays. BMC Genomics 20, 549. 10.1186/s12864-019-5864-1 31307398 PMC6632033

[B91] Porter J. J. Heil C. S. Lueck J. D. (2021). Therapeutic promise of engineered nonsense suppressor tRNAs. Wiley Interdiscip. Rev. RNA 12 (4), e1641. 10.1002/wrna.1641 33567469 PMC8244042

[B92] PR Newswire (2020). Yumanity Therapeutics and Proteostasis Therapeutics announce merger agreement [press release]. Boston, MA: PR Newswire. Available from: https://www.prnewswire.com/news-releases/yumanity-therapeutics-and-proteostasis-therapeutics-announce-merger-agreement-301116803.html .

[B93] PR Newswire (2022). SpliSense initiates phase 1/2 study of SPL84, RNA-based therapy, for the treatment of cystic fibrosis [press release]. Jerusalem, Israel: PR Newswire. Available from: https://www.prnewswire.com/news-releases/splisense-initiates-phase-12-study-of-spl84-rna-based-therapy-for-the-treatment-of-cystic-fibrosis-301702837.html .

[B94] Ramachandran S. Karp P. H. Osterhaus S. R. Jiang P. Wohlford-Lenane C. Lennox K. A. (2013). Post-transcriptional regulation of cystic fibrosis transmembrane conductance regulator expression and function by microRNAs. Am. J. Respir. Cell. Mol. Biol. 49 (4), 544–551. 10.1165/rcmb.2012-0430OC 23646886 PMC3824042

[B95] Rauscher R. Bampi G. B. Guevara-Ferrer M. Santos L. A. Joshi D. Mark D. (2021). Positive epistasis between disease-causing missense mutations and silent polymorphism with effect on mRNA translation velocity. Proc. Natl. Acad. Sci. U. S. A. 118, e2010612118. 10.1073/pnas.2010612118 33468668 PMC7848603

[B96] Sanderlin E. J. Keenan M. M. Mense M. Revenko A. S. Monia B. P. Guo S. (2022). CFTR mRNAs with nonsense codons are degraded by the SMG6-mediated endonucleolytic decay pathway. Nat. Commun. 13 (1), 2344. 10.1038/s41467-022-29935-9 35487895 PMC9054838

[B97] Sasaki S. Sun R. Bui H. H. Crosby J. R. Monia B. P. Guo S. (2019). Steric inhibition of 5' UTR regulatory elements results in upregulation of human CFTR. Mol. Ther. 27 (10), 1749–1757. 10.1016/j.ymthe.2019.06.016 31351782 PMC6822282

[B98] Sauna Z. E. Kimchi-Sarfaty C. Ambudkar S. V. Gottesman M. M. (2007). Silent polymorphisms speak: How they affect pharmacogenomics and the treatment of cancer. Cancer Res. 67 (20), 9609–9612. 10.1158/0008-5472.CAN-07-2377 17942888

[B99] Sharma N. Evans T. A. Pellicore M. J. Davis E. Aksit M. A. McCague A. F. (2018). Capitalizing on the heterogeneous effects of CFTR nonsense and frameshift variants to inform therapeutic strategy for cystic fibrosis. PLoS Genet. 14 (11), e1007723. 10.1371/journal.pgen.1007723 30444886 PMC6267994

[B100] Sharma J. Du M. Wong E. Mutyam V. Li Y. Chen J. (2021). A small molecule that induces translational readthrough of CFTR nonsense mutations by eRF1 depletion. Nat. Commun. 12 (1), 4358. 10.1038/s41467-021-24575-x 34272367 PMC8285393

[B101] Shoemaker C. J. Green R. (2012). Translation drives mRNA quality control. Nat. Struct. Mol. Biol. 19 (6), 594–601. 10.1038/nsmb.2301 22664987 PMC4299859

[B102] Simms C. L. Thomas E. N. Zaher H. S. (2017). Ribosome-based quality control of mRNA and nascent peptides. Wiley Interdiscip. Rev. RNA 8, 8. 10.1002/wrna.1366 PMC5116004 27193249

[B103] Singh R. N. Ottesen E. W. Singh N. N. (2020). The first orally deliverable small molecule for the treatment of spinal muscular atrophy. Neurosci. Insights 15, 2633105520973985. 10.1177/2633105520973985 33283185 PMC7691903

[B104] Smith E. Dukovski D. Shumate J. Scampavia L. Miller J. P. Spicer T. P. (2021). Identification of compounds that promote readthrough of premature termination codons in the CFTR. SLAS Discov. 26 (2), 205–215. 10.1177/2472555220962001 33016182 PMC7838340

[B105] Sosnay P. R. Siklosi K. R. Van Goor F. Kaniecki K. Yu H. Sharma N. (2013). Defining the disease liability of variants in the cystic fibrosis transmembrane conductance regulator gene. Nat. Genet. 45 (10), 1160–1167. 10.1038/ng.2745 23974870 PMC3874936

[B106] Sui H. Xu X. Su Y. Gong Z. Yao M. Liu X. (2022). Gene therapy for cystic fibrosis: Challenges and prospects. Front. Pharmacol. 13, 1015926. 10.3389/fphar.2022.1015926 36304167 PMC9592762

[B107] Sultan S. Rozzi A. Gasparello J. Manicardi A. Corradini R. Papi C. (2020). A peptide nucleic acid (PNA) masking the miR-145-5p binding site of the 3'UTR of the cystic fibrosis transmembrane conductance regulator (CFTR) mRNA enhances CFTR expression in calu-3 cells. Molecules 25, 1677. 10.3390/molecules25071677 32260566 PMC7181265

[B108] SYMDEKO (2020). SYMDEKO [prescribing information]. Boston, MA: Vertex Pharmaceuticals Incorporated. Available from: https://www.symdekohcp.com/ .

[B109] The Clinical and Functional TRanslation of CFTR (CFTR2) (2021). US CF foundation. Baltimore, MD: Johns Hopkins University & The Hospital for Sick Children. Available from: https://cftr2.org/ .

[B110] TRIKAFTA (2021). TRIKAFTA [prescribing information]. Boston, MA: Vertex Pharmaceuticals Incorporated. Available from: https://www.trikaftahcp.com/ .

[B111] Vaz-Drago R. Custódio N. Carmo-Fonseca M. (2017). Deep intronic mutations and human disease. Hum. Genet. 136 (9), 1093–1111. 10.1007/s00439-017-1809-4 28497172

[B112] Veit G. Oliver K. Apaja P. M. Perdomo D. Bidaud-Meynard A. Lin S. T. (2016). Ribosomal stalk protein silencing partially corrects the df508-CFTR functional expression defect. PLoS Biol. 14 (5), e1002462. 10.1371/journal.pbio.1002462 27168400 PMC4864299

[B113] Venturini A. Borrelli A. Musante I. Scudieri P. Capurro V. Renda M. (2021). Comprehensive analysis of combinatorial pharmacological treatments to correct nonsense mutations in the CFTR gene. Int. J. Mol. Sci. 22, 11972. 10.3390/ijms222111972 34769402 PMC8584557

[B114] Viart V. Bergougnoux A. Bonini J. Varilh J. Chiron R. Tabary O. (2015). Transcription factors and miRNAs that regulate fetal to adult CFTR expression change are new targets for cystic fibrosis. Eur. Respir. J. 45 (1), 116–128. 10.1183/09031936.00113214 25186262

[B115] Wen J. D. Lancaster L. Hodges C. Zeri A. C. Yoshimura S. H. Noller H. F. (2008). Following translation by single ribosomes one codon at a time. Nature 452 (7187), 598–603. 10.1038/nature06716 18327250 PMC2556548

[B116] Yi Z. Sanjeev M. Singh G. (2021). The branched nature of the nonsense-mediated mRNA decay pathway. Trends Genet. 37 (2), 143–159. 10.1016/j.tig.2020.08.010 33008628 PMC7854845

[B117] Yoshimura K. Nakamura H. Trapnell B. C. Dalemans W. Pavirani A. Lecocq J. P. (1991). The cystic fibrosis gene has a "housekeeping"-type promoter and is expressed at low levels in cells of epithelial origin. J. Biol. Chem. 266 (14), 9140–9144. 10.1016/s0021-9258(18)31562-x 1709163

[B118] Zarrilli F. Amato F. Morgillo C. M. Pinto B. Santarpia G. Borbone N. (2017). Peptide nucleic acids as miRNA target protectors for the treatment of cystic fibrosis. Molecules 22, 1144. 10.3390/molecules22071144 28698463 PMC6152032

[B119] Zielenski J. Rozmahel R. Bozon D. Kerem B. Grzelczak Z. Riordan J. R. (1991). Genomic DNA sequence of the cystic fibrosis transmembrane conductance regulator (CFTR) gene. Genomics 10 (1), 214–228. 10.1016/0888-7543(91)90503-7 1710598

